# Prevalence and Genotype Distribution of High-Risk HPV Genotypes Among Women in Greece: A Retrospective Analysis of 3500 Women

**DOI:** 10.3390/cancers17081267

**Published:** 2025-04-09

**Authors:** Dimitris Tsakogiannis, Eleni Zografos, Lamprini Tzioga, Constantinos G. Zografos, Flora Zagouri, Garyfalia Bletsa

**Affiliations:** 1 Research Center, Hellenic Anticancer Institute, 10680 Athens, Greece; 2 Department of Clinical Therapeutics, General Hospital Alexandra, 11528 Athens, Greece; el_zogra@hotmail.com (E.Z.); florazagouri@yahoo.co.uk (F.Z.); 3 A’ Surgery Department, Laiko General University Hospital of Athens, 11527 Athina, Greece; koszogra92@hotmail.com

**Keywords:** cervical cancer, HR-HPVs, persistent infection, HPV genotyping, prevention, HPV vaccines, cervical cancer screening, HPV16, infection rate, HPV distribution

## Abstract

**Background/Objectives**: Persistent infection with high-risk (HR) HPV genotypes is the main risk factor for the development of cervical cancer. The present analysis provides recent trends on HR-HPV infection rates and the distribution of HR-HPV genotypes among 3500 Greek women between 2021 and 2023. **Methods**: The detection of HR-HPVs and the specific identification of HPV16 and HPV18 were conducted using the Roche Cobas 4800 HPV assay. The genotyping of 12 other HR-HPV genotypes was performed through the Nested Multiplex PCR methodology (NMPCR). **Results:** The overall infection rate was 8.8% with the most prevalent HR-HPV genotype being HPV16 followed by HPV31, HPV66, HPV56, HPV51, HPV58, HPV45, HPV18, HPV68, HPV59, HPV52, HPV35, HPV39, and HPV33. Among HR-HPV-positive cases the prevalence of single, double, triple, and quadruple infections was 73.9%, 19.9%, 5.5%, and 0.7%, respectively. Age-specific analysis showed that the HR-HPV infection rate was higher in the age group of 31–35 years (25.5%) and it was estimated that multiple infections occur more often in younger women. Notably, the distribution of HR-HPV genotypes varies among different age groups. It is proposed that HPV16, HPV31, HPV56, and HPV66 may show an increased possibility of establishing long-term infections in Greek women over 36 years old. **Conclusions:** The high rates of specific HR-HPVs which are not included in the prophylactic vaccines underlines the significance of constant surveillance of circulating HPVs in the Greek population.

## Simple Summary

Prevention programs have successfully reduced cervical cancer incidences. However, cervical disease remains a public health issue. Long-term infection with high-risk HPV genotypes is the main risk factor for the onset of cervical cancer. Therefore, epidemiological studies concerning high-risk HPV distribution are important for the development of effective prevention strategies to better address cervical cancer burden. The present analysis provides current epidemiological data regarding high-risk HPV infection rates and the distribution of HR-HPV genotypes in women in Greece. The outcomes from the present analysis highlight the importance of HPV testing and vaccination in the Greek population. It is anticipated that the differences observed in high-risk HPV prevalence among various geographic regions and even among different age groups reflect the need to design novel and population-specific prevention tools in order to further eliminate cervical cancer in the future.

## 1. Introduction

Human papillomaviruses (HPVs) are members of the Papillomaviridae family, comprising a heterogeneous group of small, non-enveloped, double-stranded DNA viruses approximately 8000 bp in size that infect the basal layer of mucosal and cutaneous epithelia [1,2]. HPV DNA is organized into three regions: the long control region (LCR), the early region encoding for the early genes E1, E2, E4, E5, E6, and E7, and the late region encoding for the late genes L1 and L2 [3,4]. Currently, over 200 HPV genotypes have been completely characterized and grouped into five genera, including *Alphapapillomaviruses* (*Alpha-PVs*), *Betapapillomaviruses* (*Beta-PVs*), *Gammapapillomaviruses* (*Gamma-PVs*), *Mupapillomaviruses* (*Mu-PVs*), and *Nupapillomaviruses* (*Nu-PVs*) [5,6]. The strong tumorigenic capacity of HPVs has led to the classification of mucosal Alpha-PVs into high-risk (HR) and low-risk (LR) genotypes. HR-HPV genotypes have high-risk features due to their carcinogenic abilities and they are responsible for the development of precancerous lesions and cervical cancer. Currently, there are 14 HR-HPV genotypes, including HPV16, -18, -31, -33, -35, -39, -45, -51, -52, -56, -58, -59, -66, and -68. Additionally, there are 12 HPVs characterized as LR HPV genotypes, including HPV6, -11, -40, -42, -43, -44, -54, -61, -70, -72, -81, and CP6108 [7]. Epidemiological evidence has shown that HR-HPV genotypes HPV16 and HPV18 are responsible for more than 70% of invasive cervical cancers worldwide [8].

The carcinogenic potential of HPVs is caused by the function of viral E6 and E7 oncoproteins. These oncoproteins promote uncontrolled cell proliferation and block apoptosis by inactivating the cell cycle control factors p53 and pRb, respectively [9,10,11,12]. In particular, the E6 protein forms a complex with E6AP (E3 ubiquitin ligase E6-associated protein) leading to the polyubiquitination of p53 through the ubiquitin-mediated degradation pathway [12,13]. On the other hand, the E7 protein binds to pRb preventing its interaction with the E2F transcription factor. The binding of E7 with pRb activates E2F, resulting in uncontrolled cell proliferation [12,13].

Persistent infection with HR-HPV genotypes is the key factor for the development and progression of cervical cancer. Moreover, HR-HPV infection is strongly associated with several other types of cancer, such as oropharyngeal, penile, vulval, anal, and vaginal cancer [14]. Cervical cancer is the fourth most common malignancy among women worldwide, with approximately 660,000 new cases diagnosed and around 350,000 women deaths in 2022 [15]. In Greece, approximately 282 cervical cancer deaths are recorded annually with around 697 new cases diagnosed each year. It was estimated that in 2020 cervical cancer was the third leading cause of cancer-related death among Greek women [16].

Although cervical screening, HPV vaccination, and cancer treatment have significantly reduced cervical cancer incidences and deaths in many developed countries, it remains a public health issue. Nowadays, the Food and Drug Administration (FDA) and the European Medicines Agency (EMA) have approved three prophylactic HPV vaccines, including the quadrivalent Gardasil that protects against HPV6, -11, -16, and -18, the bivalent Cervarix which provides immunity against HPV16 and HPV18, and the nine-valent Gardasil 9 that protects against HPV6, -11, -16, -18, -31, -33, -45, -52, and -58 [17]. Considering cervical screening, many countries are transitioning from cytology-based tests to primary HPV testing, as HPV testing is considered more sensitive for detecting cancer precursors and enables risk stratification [18,19,20,21]. Currently, molecular methodologies based on the detection of HR-HPV DNA are widely used in laboratories and are considered the gold standard for the diagnosis of HPV infection [21]. The American Cancer Society recommends that women aged 25 to 65 should undergo a primary HPV test every 5 years. Additionally, if primary HPV testing is not accessible, cervical screening may be conducted with either a co-testing of an HPV test along with a Papanicolaou test (Pap test) every 5 years or a Pap test alone every 3 years [22]. The American Society for Colposcopy and Cervical Pathology (ASCCP; 2019) proposes that women with a positive test for HPV16 and/or HPV18 infection should be referred immediately for colposcopy [23].

Global data show that the distribution of HR-HPV genotypes varies among different populations and regions [24,25,26,27]. It has also been suggested that epidemiological outcomes regarding HR-HPV prevalence are crucial for designing successful prevention strategies to better control cervical cancer in a specific population. Towards this end, a retrospective analysis was conducted to examine the infection rate and prevalence of HR-HPVs among Greek women who underwent cervical cancer screening at the Hellenic Anticancer Institute from 2021 to 2023. The purpose of this analysis was to provide data on HR-HPV prevalence in Greece in order to better understand current trends of viral infection among Greek women.

## 2. Materials and Methods

### 2.1. Samples Collection for HR-HPV Screening

The study enrolled 3500 women from Greece who underwent HR-HPV screening at the Hellenic Anticancer Institute between 2021 and 2023. The diagnostic center selected the most sensitive screening method—a high-risk HPV DNA test with high accuracy—for the prevention and early diagnosis of cervical cancer. The age range of the examined women was between 30 and 50 years old and none of them had ever received an HPV vaccine due to the fact that when the participants were in their teens, vaccination had not yet been formally introduced in Greece. A thorough medical history was obtained, and comorbidities or prior HPV infection was not an exclusion criterion. The samples were collected using a cytobrush and immediately transferred into a vial of PreservCyt transport medium. Subsequently, the samples were transported to the laboratory for analysis. All patients signed an informed consent form and the study was approved from the research committee of the institute (approval number 14432/05-12-2019). Colposcopies were recommended for women who tested positive for HPV16 and HPV18, and a gynecological examination and retesting after a year were advised for the women infected by the remaining HR-HPV genotypes.

### 2.2. HR-HPV Screening with Roche Cobas 4800 HPV Test

In the present analysis the collected specimens were examined using the Roche Cobas 4800 HPV assay following the manufacturer’s instructions (Roche, Molecular Systems, Pleasanton, CA, USA). The Cobas HPV test is a fully automated Real-Time PCR DNA amplification assay that was approved by the Food and Drug Administration (FDA) in 2011 and developed to improve clinical use. This method is designed to identify HPV16, HPV18, and a pool of 12 other HR-HPV genotypes, including HPV31, -33, -35, -39, -45, -51, -52, -56, -58, -59, -66, and -68 [19]. Briefly, DNA extraction is carried out through a fully automated sample preparation procedure using the Cobas X 480 instrument (Roche, Molecular Diagnostics, Pleasanton, CA, USA). After DNA extraction, the specimens are transferred to the Cobas Z 480 analyzer for Real-Time PCR amplification. The Real-Time PCR enables the amplification of a highly conserved region of the L1 gene approximately 200 bp in size, while fluorescent probes facilitate the individual detection of HPV16, HPV18, and the pool detection of the 12 other HR-HPV genotypes [21]. This methodology also utilizes β-globin as an internal control (330 bp amplicon in size). Finally, the results are analyzed using software provided with the Roche Cobas 4000 HPV assay [21].

### 2.3. NMPCR for the Identification of 12 HR-HPV Genotypes

Cervical specimens that were found to be positive for other HR-HPV genotypes were further analyzed to specifically identify HPV31, -33, -35, -39, -45, -51, -52, -56, -58, -59, -66, and -68. HPV genotyping of the 12 HR-HPVs was carried out using the Nested Multiplex PCR (NMPCR) protocol as previously described [28]. The assay involved a two-step process. The first step consisted of a first-step PCR using consensus primers (GP-E6-3F, GP-E7-5B, GP-E7-6B) targeting the E6/E7 region. Subsequently, a second-step NMPCR was performed using the PCR products from the first round of amplification reaction as a template along with type-specific primer sets arranged in different primer mixtures. Each sample underwent three different NMPCRs, each utilizing a specific primer mixture. In particular, the primer mixture 1 identified HPV31, -45, and -59, the primer mixture 2 identified HPV33, -58, -52, -56, and -35, and the primer mixture 3 identified HPV68, -39, -51, and -66.

Briefly, the genomic DNA retrieved from the sample preparation process of the Cobas 4800 HPV assay was used as a template for the first-round PCR. The amplification reaction was performed in a final volume of 50 μL. The PCR mixture consisted of 15 pmol of each consensus primer, a 10 X PCR buffer (KapaTaq DNA Polymerase, Roche), containing 1.5 mM MgCl_2_, 0.2 mM from each dNTP (KapaTaq DNA Polymerase, Roche), and 1 U of thermostable DNA polymerase (KapaTaq DNA Polymerase, Roche). The cycling conditions were as follows: 40 cycles of 1 min at 94 °C, 1 min at 40 °C, and 2 min at 72 °C. The first cycle was proceeded by a 4 min denaturation step at 94 °C and the final cycle was followed by a 10 min elongation step at 72 °C. Subsequently, 2 μL of the PCR product was used as a template for NMPCR. Briefly, each NMPCR was carried out in a final volume of 50 μL. Each reaction mixture comprised 15 pmol of each type-specific primer, the 10 X PCR buffer (KapaTaq DNA Polymerase, Roche), containing 1.5 mM MgCl_2_, 0.2 mM from each dNTP (KapaTaq DNA Polymerase, Roche), and 1 U of thermostable DNA polymerase (KapaTaq DNA Polymerase, Roche). The cycling conditions were as follows: 35 cycles of 30 s at 94 °C, 30 s at 56 °C, and 45 s at 72 °C. The first cycle was proceeded by a 4 min denaturation step at 94 °C and the final cycle was followed by a 4 min elongation step at 72 °C.

The identification of HPV genotypes was conducted by monitoring the length of sizes of NMPCR amplification products in gel electrophoresis. Before gel electrophoresis, ten μL of amplicons were mixed with Midori Green direct stain (Nippon Genetics, Europe GmbH) and electrophoresed in a 2% agarose gel in Tris-borate-EDTA buffer using a 50 bp DNA ladder (Fast Gene 50 bp DNA marker, Nippon Genetics, Europe GmbH) as a molecular weight marker.

### 2.4. Statistical Analysis

The relationship between HR-HPV infection rates and age groups was determined though the chi-squared test, using GraphPad Prism 6 (GraphPad software, La Jolla, CA, USA). *p*-values were considered as statistically significant at the 0.05 cut-off level.

## 3. Results

### 3.1. The Prevalence of HR-HPV Infection in 3500 Women

**Figure 1:**
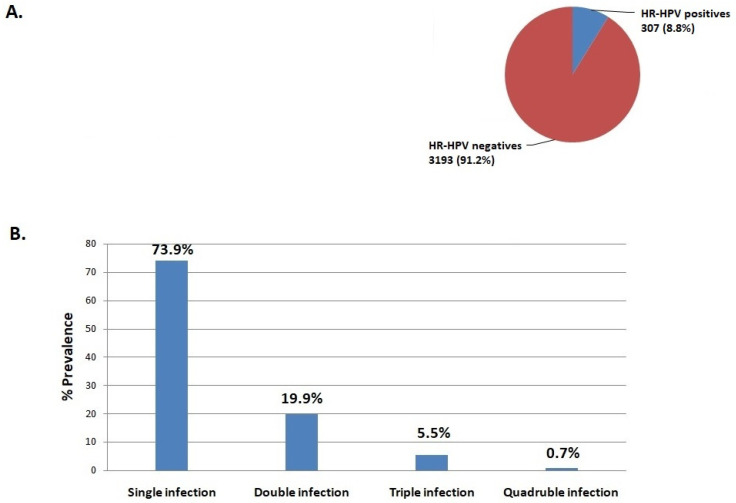
(**A**) The prevalence of HR-HPV genotypes in the 3500 examined women from the Greek population. (**B**) The distribution of single, double, triple, and quadruple infections among the HR-HPV-positive samples.

**Figure 2:**
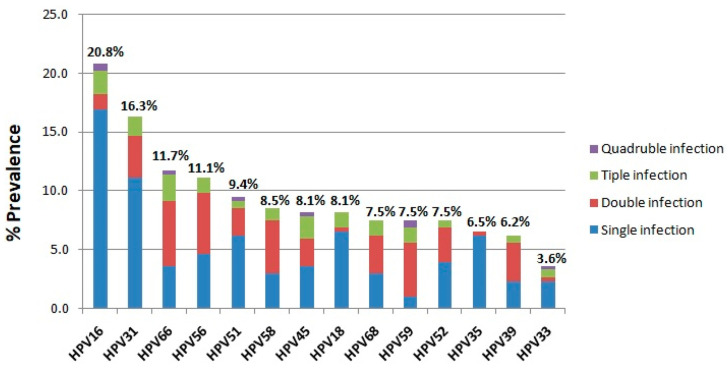
The prevalence of different HR-HPV genotypes among the HR-HPV-positive samples.

In the present analysis, a total of 3500 women from Greece between 2021 and 2023 were examined for HR-HPV infection. The age of the women examined ranged from 30 to 50 years with an average age of 42.5 ± 5.7 years. HR-HPV infection was detected in 307 of the 3500 women screened, corresponding to an overall infection rate of 8.8% (Figure 1A). According to the number of HR-HPV genotypes found in the HPV-positive specimens, the most common pattern detected was single infection with a prevalence of 73.9% (227/307), followed by double infection (19.9%, 61/307), triple infection (5.5%, 17/307), and quadruple infection (0.7%, 2/307) (Figure 1B). In addition, the most prevalent HR-HPV genotype among the HPV-positive samples was HPV16 (20.8%), followed by HPV31 (16.3%), HPV66 (11.7%), HPV56 (11.1%), HPV51 (9.4%), HPV58 (8.5%), HPV45 (8.1%), HPV18 (8.1%), HPV68 (7.5%), HPV59 (7.5%), HPV52 (7.5%), HPV35 (6.5%), HPV39 (6.2%), and HPV33 (3.6%) (Figure 2). The prevalence of different HR-HPV genotypes in single and multiple infections was further analyzed. Our results showed that HPV16, -31, -18, -51, -35, -52, -45, and -33 are more frequently detected in single HR-HPV infections accounting for 16.9%, 11.1%, 6.5%, 6.2%, 6.2%, 3.9%, 3.6%, 2.3%, respectively, while HPV66, -56, -58, -59, -68, and -39 are more frequently found in double infections accounting for 5.5%, 5.2%, 4.6%, 4.6%, 3.3%, and 3.3%, respectively (Figure 2). Triple and quadruple infections were relatively rare (Figure 2).

### 3.2. The Overall Prevalence of HR-HPV Infection in the Different Age Groups

**Figure 3:**
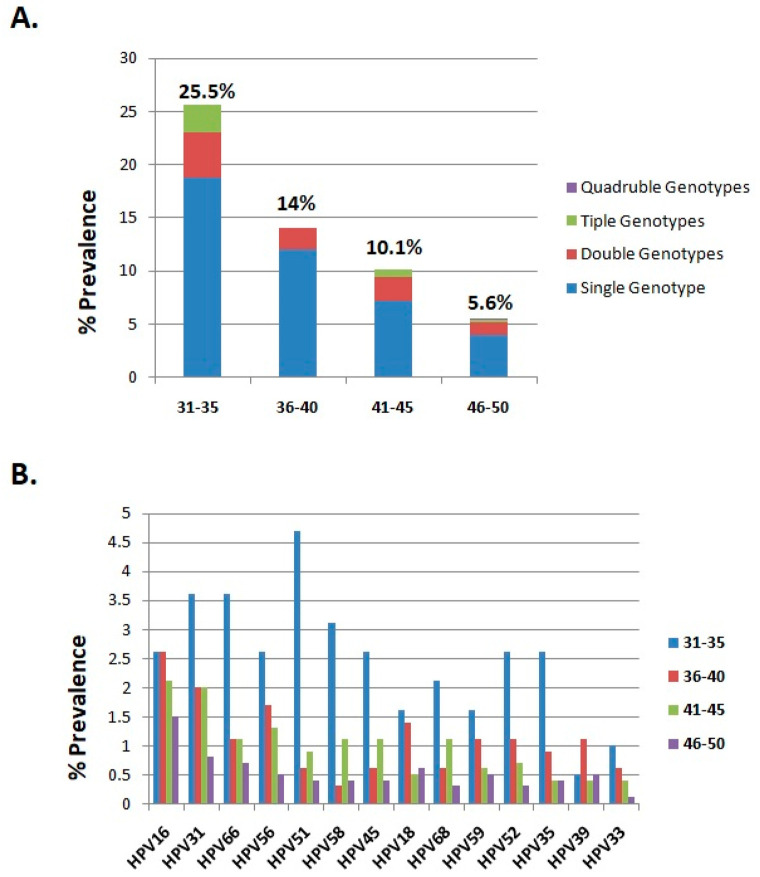
(**A**) The distribution of single, double, triple, and quadruple infections among the age groups. (**B**) The prevalence of different HR-HPV genotypes among the age groups.

The distribution of HR-HPV genotypes among different age groups is presented in Table 1. The participants were divided into three groups according to age (31–35, 36–40, 41–50 years). Among the 3500 examined women the largest proportion belonged to the 46–50 age group (57.3%, 2006/3500) followed by the 41–45 age group (27.2%, 951/3500), the 36–40 age group (10%, 351/3500), and the 31–35 age group (5.5%, 192/3500) (Table 1). The highest infection rate was recorded in the 31–35 age group (25.5%, 49/192), followed by the 36–40 age group (14%, 49/351), the 41–45 age group (10.1%, 96/951), and the 46–50 age group (5.6%, 113/2006) (Figure 3A). According to the present analysis the difference observed between the 31–35 age group and the other age cohorts was considered statistically significant (chi-squared test; 71.22, *p* < 0.0001). Moreover, it was revealed that single infection was the most common viral infection among the age groups (Figure 3A). Interestingly, a higher prevalence of multiple infections was found in the 31–35 age group (6.8%), while the proportion of multiple infections in the 36–40, 41–45, and 46–50 age groups was 2%, 2.9%, and 1.6%, respectively. The association of multiple infections with younger ages was considered as statistically significant (chi-squared test; 6.456, *p* < 0.0001).

Furthermore, differences were detected among the age groups in the top four HPV genotypes with the highest infection rates. In particular, the most prevalent HPV genotype in the 31–35 age group was HPV51 followed by HPV31, HPV66, and HPV58. In contrast, in the 36–40, 41–45, and 46–50 age groups the HR-HPV genotypes HPV16, HPV31, HPV56, and HPV66 were found to be the predominant viruses (Table 1, Figure 3B).

### 3.3. The Prevalence of HR-HPV Genotypes Between 2021 and 2023

**Figure 4:**
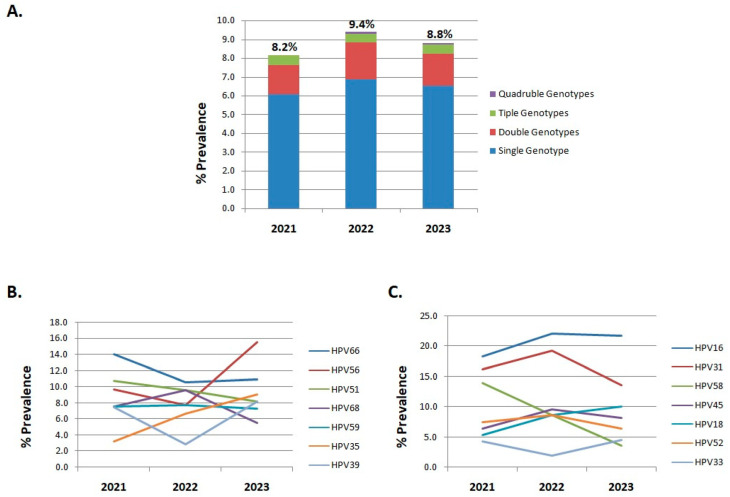
(**A**) The overall HR-HPV infection rate in 2021, 2022, and 2023. (**B**) The prevalence of HR-HPV genotypes in 2021, 2022, and 2023 that are not covered by HPV vaccines. (**C**) The prevalence of HR-HPV genotypes in 2021, 2022, and 2023 that are covered by HPV vaccines.

The prevalence of different HR-HPV genotypes between 2021 and 2023 is presented in Table 2. Our results indicate that the overall infection rate in 2021 was 8.2%, in 2022 the infection rate was 9.4%, and in 2023 the HR-HPV infection rate was estimated at 8.8% (Table 2, Figure 4A). Additionally, the most frequent pattern was single infection followed by double, triple, and quadruple infection in all age groups (Table 2, Figure 4A). The distribution of HR-HPV genotypes in HPV positive cases varied slightly among the different age groups. In particular, in 2021 and 2022 the three most common HR-HPV genotypes with the highest infection rates were HPV16, HPV31, and HPV66, while in 2023 the three most common HR-HPV genotypes were HPV16, HPV56, and HPV31 (Table 2). It is noteworthy that over the years, HPV56, which is not covered by the HPV nine-valent vaccine, demonstrated an upward trend (Table 3, Figure 4B). Specifically, the prevalence of HPV56 in HPV-positive women increased from 9.7% in 2021 to 15.5% in 2023, providing a 1.6-fold increase with its infection rate ranking second among the infected women in 2023 (Table 3, Figure 4B). Further analysis of the distribution of HR-HPV genotypes that are not covered by the HPV nine-valent vaccine showed modifications throughout the years. Specifically, HPV35 exhibited a 2.8-fold increase between 2021 and 2023, thus ranking sixth among the infected women in 2023 (Table 3, Figure 4B). Finally, the distribution of HPV genotypes HPV66, -51, and -68 seems to decrease between 2021 and 2023, while HPV59 and -39 appear to remain rather stable (Table 3, Figure 4B).

The prevalence of HR-HPV genotypes that are covered by the HPV nine-valent vaccine revealed fluctuations over the examined years, as well (Table 3, Figure 4C). In particular, the prevalence of HPV58 decreased from 14% in 2021 to 3.6% in 2023, providing a 3.9-fold decrease (Table 3, Figure 4C). Notably, the infection rate of HPV18 increased from 5.4% in 2021 to 10% in 2023, showing a 1.85-fold increase with its infection rate ranking fifth among the infected women in 2023. Finally, the infection rate of HPV16 remained consistently higher than the other HR-HPV genotypes during the examined years, while the prevalence of HPV31, -45, -52, and -33 is relatively low (Table 3, Figure 4C).

## 4. Discussion

Cervical cancer is the first type of cancer that could potentially be eliminated in the future through prevention programs. The strategies implemented could provide valuable insights for future programs to combat other cancers [29]. Today, cervical cancer incidence and mortality rates vary considerably by country and region. Data from the WHO global assessment for 2020 shows that 10% of all cervical cancer cases are detected in Europe, which ranks third after Asia (58%) and Africa (20%) [30]. Europe also has the lowest mortality rate in the world [30]. Currently, HPV vaccination and HPV screening programs are the most effective tools for eliminating the burden of cervical cancer [31]. Data on the prevalence of HR-HPV genotypes in different geographic areas are crucial for accurate cervical cancer screening and HPV vaccination strategies to better target cervical cancer in specific populations. The current analysis provides up-to-date evidence on HR-HPV infection rates and the distribution of different HR-HPV genotypes among women in Greece who have undergone annual cervical cancer screening.

HR-HPV genotypes are cancer-causing viruses. An HR-HPV infection that is not eliminated by the immune system is responsible for the development of cervical cancer (see Supplementary Materials). The worldwide HPV infection rate varies among different population groups and geographic regions. The highest HPV infection rates were observed in North America (54,6%) and the United States (40%), followed by sub-Saharan Africa (24%) and China (16,02%) [32,33,34,35,36]. In Europe, HR-HPV infection rates differ across regions. Eastern Europe has an average infection rate of 21.4%, followed by Southern Europe (17.2%) and Western Europe (9%) [37]. In the present analysis, the overall rate of HR-HPV infection in the Greek population was 8.8%, consistent with rates reported in Western Europe [37]. Previous analyses conducted in Greece between 2007 and 2014 showed infection rates between 5.8% to 39.5% [37,38,39,40,41,42]. The discrepancies in the data may be due to differences in the time periods analyzed and the varying sensitivity of the detection methods used in each study.

Another aspect of the present analysis was to investigate the prevalence of single and multiple infections in the Greek population. The impact of multiple infections on the development of cervical cancer requires further investigation as controversial results have emerged. In particular, it has been suggested that multiple HPV infections may promote intergenotypic competition and immune responses, thus preventing the development of severe dysplasia [43]. In contrast, other studies have implied that multiple viral infections more efficiently lead to persistent infections and the development of cervical cancer [44,45,46]. One possible explanation could be the synergistic effect between different HPV genotypes that more effectively evade the host immune response and thus promote the establishment of long-term HPV infections [44,45,46]. In the present analysis, a single infection was found to be more prevalent in HPV-positive Greek women (73.9%), while multiple HR-HPV infections were found in 26.1% of the positive samples analyzed. These results are consistent with previous findings [36,47,48]. Further analysis of single and multiple infections revealed that the trend towards multiple infections is increasing in younger women. This observation is in line with previous findings, showing that multiple infections are more common in younger women [36,49]. However, the impact of multiple HR-HPV infections in young Greek women needs to be further investigated and followed up to assess whether these women are at higher risk of developing severe precancerous lesions and cervical cancer in the future.

Additional analysis of the age groups revealed that the HR-HPV infection rate was higher in the 31–35 age group (25.5%) and decreased in the other age groups, with the lowest infection rate observed in the 46–50 age group (5.6%). The higher prevalence of HR-HPV infection in younger women was found to be statistically significant (chi-squared test; 71.22, *p* < 0.0001). Previous analyses in European, Chinese, and American populations showed similar results, as HR-HPV prevalence was higher in women younger than 35 years, whereas it decreased in older age groups [36,47,50,51]. Interestingly, in various analyses the lower rate is observed from the age of 45 years, which is consistent with our results [36,47,50,51]. Considering that HPV is regarded as the most common sexually transmitted infection, it was concluded that the higher infection rates and increased prevalence of multiple HR-HPV infections in younger women may be related to the characteristics of sexual behavior commonly observed in younger people, including frequent sexual activity and instability of sexual partners.

Previous epidemiological studies have revealed that the prevalence of different HPV genotypes varies among different regions, with HPV16 and HPV18 being the most prevalent HR-HPV genotypes in cervical cancer cases worldwide [52]. A recent meta-analysis estimated that HPV16 and HPV18 are detected in 49.8% and 45.3% of cervical adenocarcinoma cases, respectively [52]. Therefore, early detection of HPV16 and HPV18 can stratify individuals at highest risk for cervical disease [21]. In the present analysis, HPV16 was the most prevalent HR-HPV genotype, detected in 1.8% of the examined women (20.8% of positive samples), while its prevalence remained high in all age groups. Additionally, the prevalence of HPV18 is rather low, as it was detected in 0.7% of the samples analyzed (8.1% of positive samples), placing it in eighth place. Previous studies have revealed that the range of HPV16 prevalence varies across European countries, with the highest rates observed in France (10.6%) and Belgium (5.6%), while the lowest rates were seen in the Netherlands (1%), Sweden (1.7%), and Norway (1.3%), [37]. Interestingly, the HPV16 infection rate recorded in the Greek population was lower than that observed in other Southern European countries, including Italy (2.1%) and Spain (2.5%) [37]. A low prevalence of HPV18 has also been recorded in Europe (1%), which is consistent with the results of the present analysis [37].

Although HPV16 is the most prevalent HR-HPV genotype worldwide, global data have shown an increased prevalence of HPV18 (1.4%), HPV53 (0.9%), and HPV31 (0.8%) following HPV16 (3.2%). In the European population, a high prevalence of HPV31 (2.3%), HPV18 (0.9%), and HPV39 (0.8%) after HPV16 (4.8%) was estimated [47]. In the current study, it was found that in the Greek population, the next most common HR-HPV genotypes after HPV16 (1.8%) were HPV31 (1.4%), HPV66 (1%), HPV56 (1%), and HPV51 (0.8%). In light of these data, it is suggested that more attention be paid to HPV66, -56, and -51 in Greece, as these genotypes are not covered by the prophylactic nine-valent HPV vaccine. Therefore, up-to-date data from different regions are essential to better understand the distribution of HR-HPV genotypes and to serve as a basis for the development and evaluation of novel multivalent vaccines specific to the region.

Further analysis of the distribution of HR-HPV genotypes in different age groups revealed that the four most prevalent HR-HPV genotypes HPV16, HPV31, HPV56, and HPV66 were consistent in women older than 36 years old. However, the prevalence of other HR-HPV genotypes was as high or higher in the 31–35 age group, including HPV51, HPV31, HPV66, and HPV58. Differences among younger and older women in HPV prevalence have already been reported, suggesting that the HR-HPV genotypes detected in older women have a higher probability of establishing persistent infections [53]. Considering that HPV infections are transient and usually resolve spontaneously within 24 months, it has been concluded that the predominant HR-HPV genotypes, including HPV16, HPV31, HPV56, and HPV66, may have a higher possibility of long-term infections in Greek women over 36 years of age [53,54]. However, further analyses are needed to test this hypothesis. Consequently, the diagnosis of HPV infection and especially the identification of specific HR-HPV genotypes in a given population is crucial for the selection of individuals at highest risk of persistent infection and development of cervical cancer. In addition, a comprehensive analysis of HPV prevalence in different age cohorts could provide valuable information for the selection of more effective prophylactic vaccines tailored to specific age groups in a population in the future [53].

Various fluctuations in the prevalence of different HR-HPV genotypes have been observed over the years. However, the duration of the current study is relatively short to evaluate the reasons for these changes and, in particular, to assess whether these changes are due to the effect of HPV vaccination in the Greek population. Therefore, further analyses are needed to draw more definite conclusions. It is important to emphasize that the increasing trend of HR-HPV genotypes HPV56 and HPV35, which are not covered by the nine-valent HPV vaccine, in the Greek population may require greater attention in the coming years.

## 5. Conclusions

In summary, the present analysis reveals current epidemiological trends regarding HR-HPV infections and the distribution of HR-HPV genotypes in Greek women. The age-specific prevalence of HR-HPV infection in the examined population reflects the importance of HPV testing and vaccination in younger women. In addition, the increasing rates of specific HR-HPV genotypes not covered by prophylactic vaccines highlight the need for continuous surveillance of circulating HPVs in the Greek population. These results suggest that the comprehensive analysis of HR-HPV prevalence is crucial for the development of population-specific preventive measures to further reduce cervical cancer burden.

## Figures and Tables

**Table 1:** HR-HPV infection rates and the distribution of different HR-HPV genotypes among the age groups 31–35 years, 36–40 years, 41–45 years, and 46–50 years.

	31–35	36–40	41–45	46–50	Total
	n (%)	n (%)	n (%)	n (%)	n (%)
**Samples**	192 (5.5)	351 (10)	951 (27.2)	2006 (57.3)	3500 (100)
**HPV infection**	49 (25.5)	49 (14)	96 (10.1)	113 (5.6)	307 (8.8)
**Single genotypes**	36 (18.8)	42 (12)	68 (7.2)	81 (4)	227 (6.5)
**Double genotypes**	8 (4.2)	7 (2)	22 (2.3)	24 (1.2)	61 (1.7)
**Triple genotypes**	5 (2.6)	0	6 (0.6)	6 (0.3)	17 (0.5)
**Quadruple genotypes**	0 (0)	0	0	2 (0.1)	2 (0.1)
**HPV genotypes**					
HPV16	5 (2.6)	9 (2.6)	20 (2.1)	30 (1.5)	64 (1.8)
HPV31	7 (3.6)	7 (2)	19 (2)	17 (0.8)	50 (1.4)
HPV66	7 (3.6)	4 (1.1)	10 (1.1)	15 (0.7)	36 (1)
HPV56	5 (2.6)	6 (1.7)	12 (1.3)	11 (0.5)	34 (1)
HPV51	9 (4.7)	3 (0.9)	9 (0.9)	8 (0.4)	29 (0.8)
HPV58	6 (3.1)	1 (0.3)	10 (1.1)	9 (0.4)	26 (0.7)
HPV45	5 (2.6)	2 (0.6)	10 (1.1)	8 (0.4)	25 (0.7)
HPV18	3 (1.6)	5 (1.4)	5 (0.5)	12 (0.6)	25 (0.7)
HPV68	4 (2.1)	2 (0.6)	10 (1.1)	7 (0.3)	23 (0.7)
HPV59	3 (1.6)	4 (1.1)	6 (0.6)	10 (0.5)	23 (0.7)
HPV52	5 (2.6)	4 (1.1)	7 (0.7)	7 (0.3)	23 (0.7)
HPV35	5 (2.6)	3 (0.9)	4 (0.4)	8 (0.4)	20 (0.6)
HPV39	1 (0.5)	4 (1.1)	4 (0.4)	10 (0.5)	19 (0.5)
HPV33	2 (1)	2 (0.6)	4 (0.4)	3 (0.1)	11 (0.3)

**Table 2:** HR-HPV infection rates and the distribution of different HR-HPV genotypes in 2021, 2022, and 2023.

	2021	2022	2023
	n (%)	n (%)	n (%)
**Samples**	1139	1108	1253
**HPV infection**	93 (8.2)	104 (9.4)	110 (8.8)
**Single genotype**	69 (6.1)	76 (6.9)	82 (6.5)
**Double genotypes**	18 (1.6)	22 (2)	21 (1.7)
**Triple genotypes**	6 (0.5)	5 (0.5)	6 (0.5)
**Quadruple genotypes**	0 (0)	1 (0.1)	1 (0.1)
**HPV genotypes**			
HPV16	17 (1.5)	23 (2.1)	24 (1.9)
HPV31	15 (1.3)	20 (1.8)	15 (1.2)
HPV66	13 (1.1)	11 (1)	12 (1.0)
HPV56	9 (0.8)	8 (0.7)	17 (1.4)
HPV51	10 (0.9)	10 (0.9)	9 (0.7)
HPV58	13 (1.1)	9 (0.8)	4 (0.1)
HPV45	6 (0.5)	10 (0.9)	9 (0.7)
HPV18	5 (0.4)	9 (0.8)	11 (0.9)
HPV68	7 (0.6)	10 (0.9)	6 (0.5)
HPV59	7 (0.6)	8 (0.7)	8 (0.6)
HPV52	7 (0.6)	9 (0.8)	7 (0.6)
HPV35	3 (0.3)	7 (0.6)	10 (0.8)
HPV39	7 (0.6)	3 (0.3)	9 (0.7)
HPV33	4 (0.4)	2 (0.2)	5 (0.4)

**Table 3:** The prevalence of different HR-HPV genotypes in 2021, 2022, and 2023 in HPV-positive samples.

	2021	2022	2023
**HPV-positive samples (n)**	93	104	110
**HPV genotypes**	**n (%)**	**n (%)**	**n (%)**
HPV16	17 (18.3)	23 (22.1)	24 (21.8)
HPV31	15 (16.1)	20 (19.2)	15 (13.6)
HPV66	13 (14)	11 (10.6)	12 (10.9)
HPV56	9 (9.7)	8 (7.7)	17 (15.5)
HPV51	10 (10.8)	10 (9.6)	9 (8.2)
HPV58	13 (14)	9 (8.7)	4 (3.6)
HPV45	6 (6.5)	10 (9.6)	9 (8.2)
HPV18	5 (5.4)	9 (8.7)	11 (10)
HPV68	7 (7.5)	10 (9.6)	6 (8.2)
HPV59	7 (7.5)	8 (7.7)	8 (7.3)
HPV52	7 (7.5)	9 (8.7)	7 (6.4)
HPV35	3 (3.2)	7 (6.7)	10 (9.1)
HPV39	7 (7.5)	3 (2.9)	9 (8.2)
HPV33	4 (4.3)	2 (1.9)	5 (4.5)

## Data Availability

Data are included in the manuscript.

## Supplementary Materials

The following supporting information can be downloaded at https://www.mdpi.com/article/10.3390/cancers17081267/s1, Figure S1: Persistent infection with HR-HPV genotypes is the main risk factor for the development of precancerous lesions and cervical cancer.
