# Supplementary figures and images for: Prevalence and Genotype Distribution of High-Risk HPV Genotypes Among Women in Greece: A Retrospective Analysis of 3500 Women
Source: Cancers (Basel). 2025 Apr 9;17(8):1267. doi: 10.3390/cancers17081267 (PMC12026139; doi:10.3390/cancers17081267)

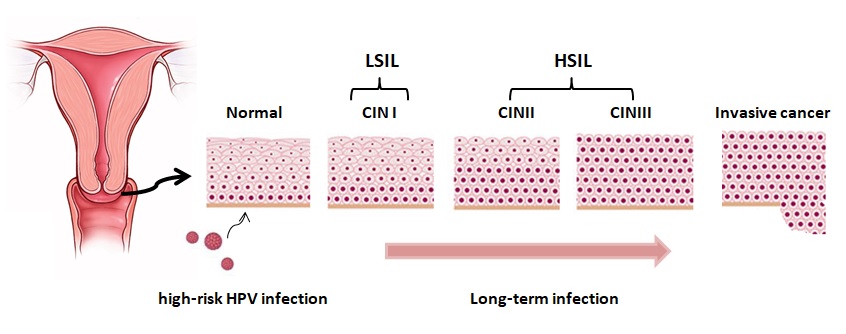

Supplement: Supplementary file 1 [file cancers-17-01267-s001.zip › Figure S1.jpg]
